# Primary cutaneous B-cell lymphoma arising from an inguinal hernioplasty scar

**DOI:** 10.1093/jscr/rjab605

**Published:** 2022-01-21

**Authors:** Ricardo Vaz-Pereira, Herculano Moreira, Ana Monteiro, Ana Melo, Clara Leal, Bruno Vieira, Francisca Freitas, Carolina Marques, João Pinto-de-Sousa

**Affiliations:** Department of General Surgery, Centro Hospitalar De Trás-Os-Montes E Alto Douro, Vila Real, Portugal; Department of General Surgery, Centro Hospitalar De Trás-Os-Montes E Alto Douro, Vila Real, Portugal; Department of General Surgery, Centro Hospitalar De Trás-Os-Montes E Alto Douro, Vila Real, Portugal; Department of General Surgery, Centro Hospitalar De Trás-Os-Montes E Alto Douro, Vila Real, Portugal; Department of General Surgery, Centro Hospitalar De Trás-Os-Montes E Alto Douro, Vila Real, Portugal; Department of General Surgery, Centro Hospitalar De Trás-Os-Montes E Alto Douro, Vila Real, Portugal; Department of General Surgery, Centro Hospitalar De Trás-Os-Montes E Alto Douro, Vila Real, Portugal; Department of General Surgery, Centro Hospitalar De Trás-Os-Montes E Alto Douro, Vila Real, Portugal; Department of General Surgery, Centro Hospitalar De Trás-Os-Montes E Alto Douro, Vila Real, Portugal

## Abstract

The primary cutaneous B-cell lymphoma (PCBCL) is a rare neoplasm. It is believed that antigenic stimulation and chronic inflammation can be the basis of pathogenesis. Here, we report a case that to our knowledge is the first patient with a presentation of a PCBCL arising from a surgical scar, in particular, an inguinal hernioplasty. The case reminds us of the importance of raising clinical suspicion for malignant neoplasms in surgical scars, in particular, after mesh placement. **MeSH terms**: ‘lymphoma, large b-cell, diffuse’, ‘skin’ and ‘cicatrix’.

## INTRODUCTION

Primary cutaneous lymphoma (PCL) is characterized by only skin involvement at the time of diagnosis. It is a rare neoplasm with an incidence of 0.5–1 per 100 000 [1]. The B-cell variant designed primary cutaneous B-cell lymphoma (PCBCL) represents only 20–25% of all PCLs [2] and is classified according to 2017 World Health Organization Criteria into three types: primary cutaneous marginal zone lymphoma, primary cutaneous diffuse large B-cell lymphoma, leg-type and primary cutaneous follicle center lymphoma (PCFCL). It is believed that the basis of pathogenesis of these lymphomas is antigenic stimulation on a skin area, which leads to cutaneous lymphoid hyperplasia, and finally lymphoma [3]. *Borrelia burgdorferi* infection and tattoos are described as antigenic stimuli [3], and two cases of PCBCL arising from a burn scar [4] and Herpes Zoster scar [5] have also been described. However, to the best of our knowledge, no case of PCBCL arising from a surgical scar has been described by searching in PubMed with the MeSH terms ‘lymphoma, large b-cell, diffuse’; ‘skin’ and ‘cicatrix’, or by the terms ‘primary cutaneous b-cell lymphoma’; ‘surgery’; ‘scar’ and ‘mesh’. Herein, we presented a rare case of a PCBCL arising from an inguinal hernioplasty scar.

## CASE REPORT

**Figure 1:**
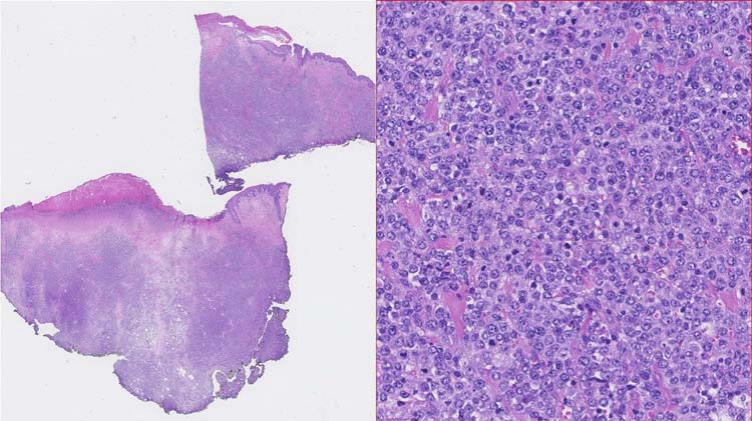
Photomicrographies. Left: Small magnification (HE) of a fragment of skin and subcutaneous tissue, with extensive epidermal ulceration and almost total occupation of the subcutaneous tissue by lymphoid tissue placed in a towel. Right: High magnification (HE ×40) with cytological detail of the lymphoid infiltrate, consisting of towels of voluminous cells with centroblastic morphology.

**Figure 2:**
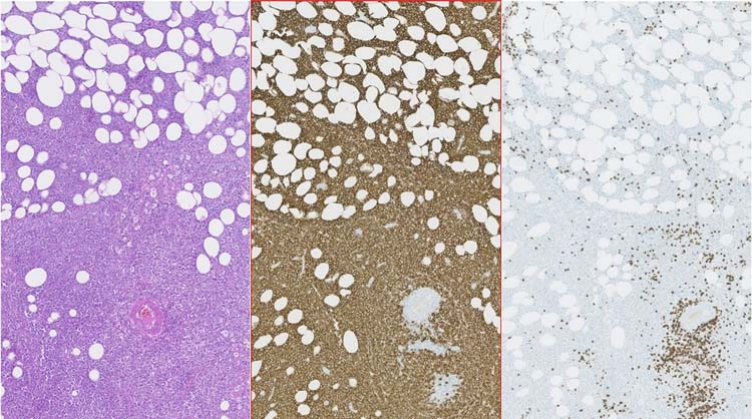
Photomicrographies. Left: HE ×5, with an area of hypodermic adipose tissue infiltrated by the neoplastic lymphoid population. Center: Immunohistochemical staining of the same area, revealing diffuse expression of CD20 by neoplastic lymphoid cells. Right: Absence of CD3 expression in the neoplastic cell population and expression only in a small amount of dispersed reactive T lymphocytes.

**Figure 3:**
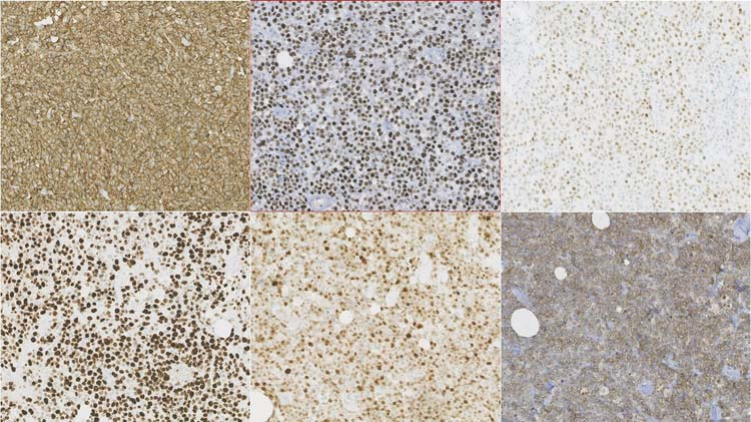
Photomicrographies. Lymphoid infiltrate immunophenotype, with a diffuse expression of CD20, bcl6, C-Myc (upper half from left to right), and Ki67, MUM1 and CD10 (lower half from left to right).

**Figure 4:**
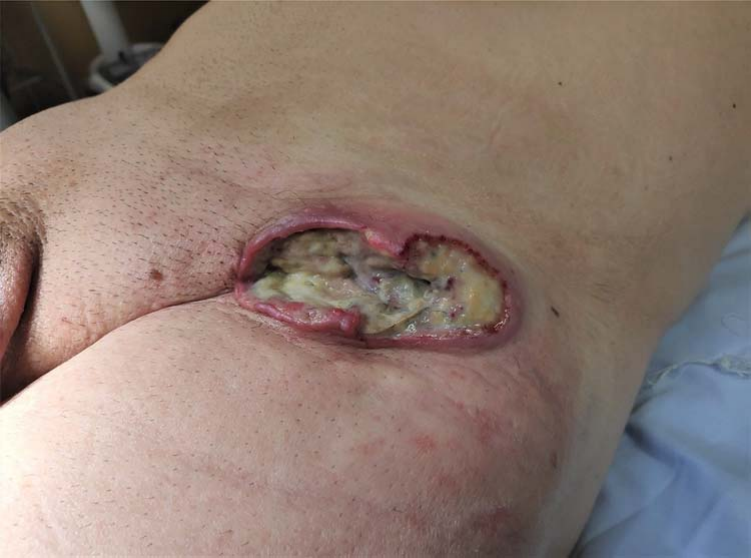
PCBCL at the site of the previous hernioplasty.

**Figure 5:**
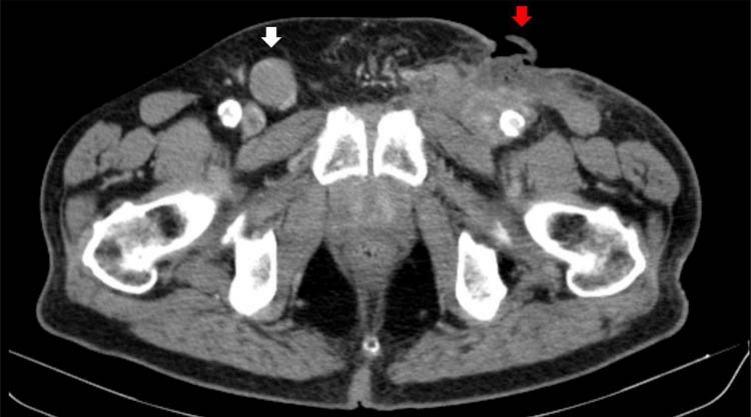
Computerized tomography image. Right inguinal adenopathy (white arrow) and PCBCL (red arrow).

A 73-year-old Caucasian man with a history of hypertension, atrial flutter and Type 2 diabetes mellitus underwent left inguinal hernia repair by Rutkow-Robbins hernioplasty in 2017 without immediate surgical or postoperative complications. In 2021, he reports the appearance of a painless swelling in the left inguinal scar. The patient was medicated with antibiotics without improvement and was sent to the General Surgery consultation with the diagnosis of inguinal mesh rejection. Upon observation by General Surgery, the patient had an ulcerated area of the medial portion of the scar, measuring 1 × 2 cm, with drainage of seropurulent content and surrounding erythema, interpreted as the external orifice of a fistulous tract. The diagnosis of late mesh rejection was maintained, and the patient underwent prosthesis removal without hernia repair. During surgery, extensive peri-mesh fibrosis was found but without apparent signs of infection. The scar was removed *en bloc* including the ulcerated area and sent for anatomopathological examination. The surgery was uneventful and the patient was discharged. Histological examination (Figs 1–3) revealed extensive involvement of the dermis and hypodermis by a high-grade non-Hodgkin lymphoproliferative process with characteristics of diffuse large-cell B lymphoma, which focally conditions epidermal ulceration. The immunohistochemical study showed diffuse staining for CD20, MUM-1 and CD10, with weak and heterogeneous staining in ~40–50% of neoplastic cells for C-MYC and 80–90% of the same population cell for Ki-67. Immunostains for CD30 were not observed and with anti-CD3 and anti-CD5 antibodies, only marking of scattered rare reactive T lymphocytes was observed. Finally, the neoplastic cell population had no staining for bcl2, but diffuse staining for bcl6. Upon reassessment, the patient had wound dehiscence with an extensive ulcerated area (Fig. 4) and was oriented to hematology. Bone marrow biopsy, myelogram and flow cytometry were normal and computerized tomography of the neck, chest, abdomen and pelvis revealed a right inguinal adenopathy (Fig. 5). The patient was staged as IIa and proposed for systemic treatment with rituximab, cyclophosphamide, doxorubicin, vincristine and prednisolone (R-CHOP).

## DISCUSSION

To our knowledge, the case is the first described PCBCL occurring on surgical scar tissue. *Borrelia burgdorferi* infection, tattoos, venous insufficiency, lymphatic stasis, burn scar and Herpes Zoster scar have already been described as triggering factors [3–5]. For the first two, it is believed that antigenic stimulation is the basis of pathogenesis. For the others, chronic inflammation and immune dysregulation may be the cause, in a process similar to the origin of the T-cell variant anaplastic large-cell lymphoma occurring in women with breast implants. On this patient, a polypropylene mesh was used, a type of plastic known to be inert, safe and widely used in surgery. Nevertheless, we believe that chronic inflammation, either by the foreign body effect of the mesh or by scarring, was the genesis of this lymphoma.

At the time of diagnosis, the patient had no signs or symptoms of extracutaneous disease, by which a PCL was considered. Findings shown by histological examination and immunohistochemistry were consistent with the PCFCL variant.

Unfortunately, we do not have images of the ulcerated lesion at the first evaluation, but initially, we believed that we were facing a case of late mesh rejection. This case reminds us of the importance of raising clinical suspicion for malignant neoplasms in surgical scars, in particular, after mesh placement, even extremely rare. In atypical presentations, a skin biopsy should be considered.

## AUTHORS’ CONTRIBUTIONS

Ricardo Vaz Pereira contributed to the conception of this article, review the literature and wrote the manuscript. Herculano Moreira, Ana Monteiro, Ana Melo, Clara Leal, Bruno Vieira, Francisca Freitas, Carolina Marques and João Pinto-de-Sousa had a special contribution in the conception and the reviewing of the article.

## CONFLICT OF INTEREST STATEMENT

The authors have no conflicts of interest to declare.

## FUNDING

The authors declare that no financial support was received.

## ETHICS STATEMENT

This manuscript is in accordance with the rules of our Institutional Ethics Committee. Written informed consent was obtained from the patient.
